# Supplementary material for: Achieving net zero greenhouse gas emissions critical to limit climate tipping risks
Source: Nat Commun. 2024 Aug 1;15:6192. doi: 10.1038/s41467-024-49863-0 (PMC11294534; doi:10.1038/s41467-024-49863-0)
Supplement: Supplementary file 1 — Supplementary Information [file 41467_2024_49863_MOESM1_ESM.pdf]

Supplementary Information: Achieving net zero greenhouse gas emissions critical to limit climate tipping risks

Tessa Möller[1,2,3,4,5,*,+], Annika Ernest Högner[3,4,5,*], Carl-Friedrich Schleussner[1,2,6], Samuel Bien[3,4,5], Niklas H. Kitzmann[3,4], Robin D. Lamboll[7], Joeri Rogelj[1,7,8], Jonathan F. Donges[3,9,10], Johan Rockström[3,5,9], Nico Wunderling[3,10,11,+]

[1] Energy, Climate and Environment Program, International Institute for Applied Systems Analysis (IIASA), Laxenburg, Austria
[2] Climate Analytics, Berlin, Germany
[3] Potsdam Institute for Climate Impact Research (PIK), Member of the Leibniz Association, Potsdam, Germany
[4] Institute of Physics and Astronomy, University of Potsdam, Potsdam, Germany
[5] Institute of Environmental Science and Geography, University of Potsdam, Potsdam, Germany
[6] Geography Department & IRI THESys, Humboldt University of Berlin, Berlin, Germany
[7] Grantham Institute for Climate Change and the Environment, Imperial College London, London, UK
[8] Centre for Environmental Policy, Imperial College London, London, UK
[9] Stockholm Resilience Centre, Stockholm University, Stockholm, Sweden
[10] High Meadows Environmental Institute, Princeton University, Princeton, NJ, USA
[11] Center for Critical Computational Studies (C³S), Goethe University Frankfurt, Frankfurt am Main, Germany
* These authors contributed equally to this study
+ Corresponding authors: moeller@iiasa.ac.at, nico.wunderling@pik-potsdam.de

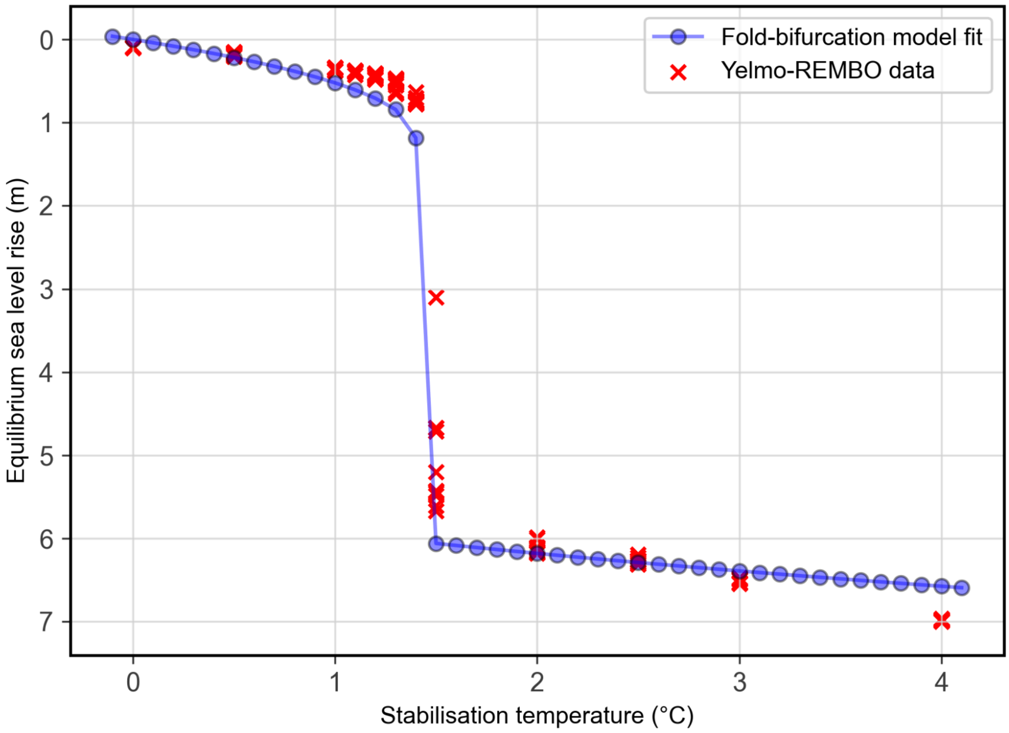

Supplementary Fig. 1 | Exemplary validation of the x^3 fold-bifurcation model against the stability diagram of the Greenland Ice Sheet (GIS). *The first terms of the model equation describe the individual tipping dynamics of a tipping element without any couplings, here with exponent $n = 3$ (see Eq. 1). The red crosses are taken from Bochow et al. 2023 Fig. 2a[1], and denote the stability landscape of the GIS found with the ice-sheet model Yelmo, coupled to the Regional Energy-Moisture Balance Orographic (REMBO) model. The blue points and line denote the fit of the x^3 fold-bifurcation model. We have performed the fit with a critical temperature of 1.49 °C and find that our model is able to qualitatively reproduce the results found by Bochow et al. in good agreement.*

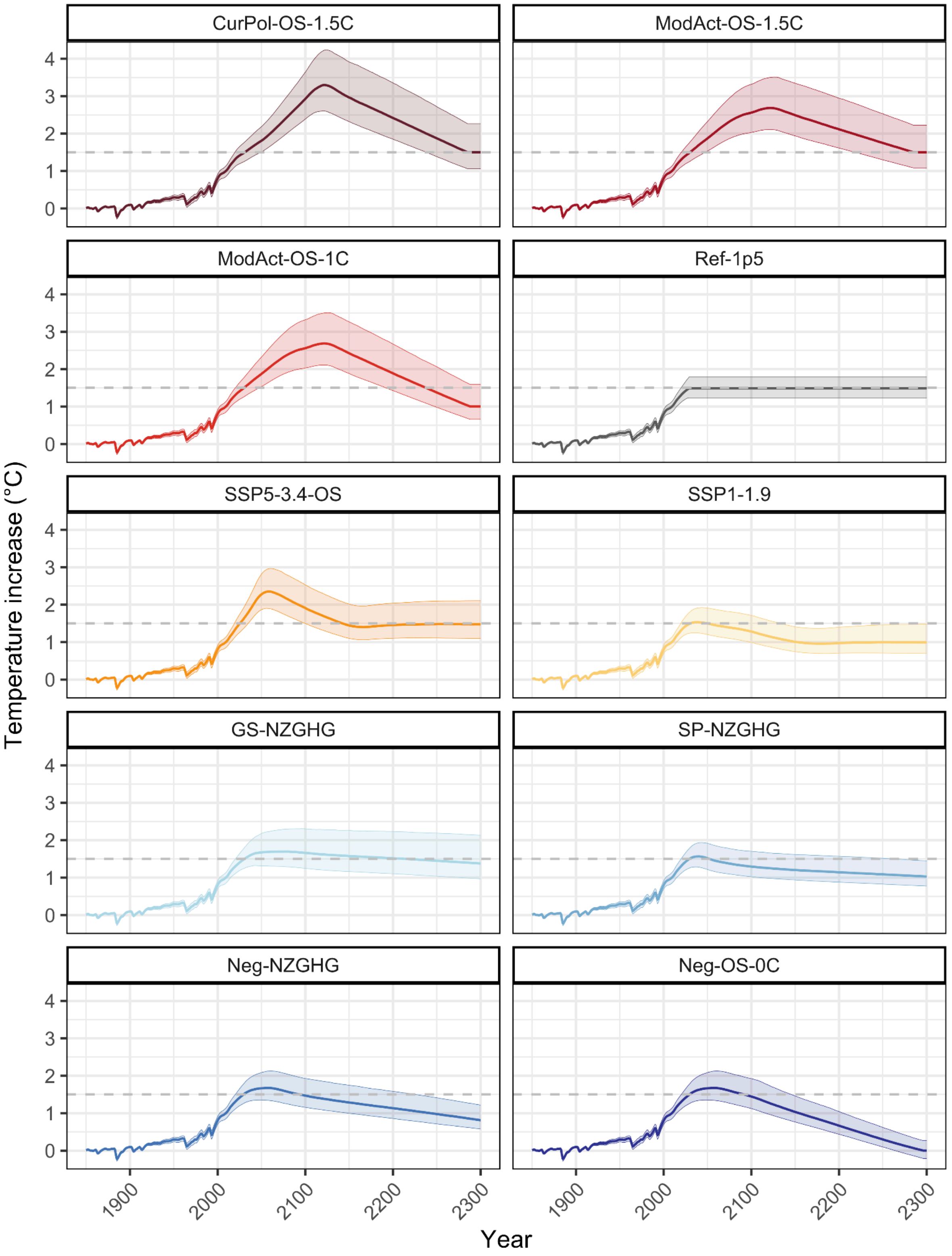

***Supplementary Fig. 2 | Temperature trajectories** for the selected PROVIDE emission scenarios from 1850 to 2300 derived with the FaIR emulator. The temperature anomaly relative to the 1850-1900 average is shown as median with the uncertainty corridor between the 10 % and 90 % quantiles.*

Tipping element	Critical temperature	Timescale (years)
GIS	0.8 - 3.0 °C	1000 - 15000
WAIS	1.0 - 3.0 °C	500 - 13000
AMAZ	2.0 - 6.0 °C	50 - 200
AMOC	1.4 - 8.0 °C	15 - 300

Supplementary Table 1 | Tipping element parameters. *Critical temperature ranges and timescales for each tipping element, taken from Armstrong McKay et al. (2022).*

Interaction	Max. link strength	Physical process
GIS → AMOC	+10	Freshwater influx
AMOC → GIS	−10	Reduced northward heat transport
GIS → WAIS	+10	Sea level rise
WAIS → GIS	+2	Sea level rise
AMOC → WAIS	+1.5	Reduced northward heat transport
AMOC → AMAZ	±4	Precipitation changes
WAIS → AMOC	±3	Changes in salinity gradient (−) and freshwater anomaly (+)

Supplementary Table 2 | Interaction links between tipping elements in the network. *Positive link strengths indicate self-amplifying feedbacks that increase the tipping risk for the receiving tipping element, negative link strengths indicate negative feedbacks that decrease the tipping risk of the receiving tipping element. Interactions with ± sign are unclear, i.e. it is not known whether they will increase or decrease the risk of tipping for the receiving tipping element. The link strengths* s_{ij} *are taken from Wunderling et al. (ESD, 2021) with a minor modification (the unclear link from AMOC to AMAZ now spans the full range from -4 to 4 to include the possibility for weak interactions) and are based on an expert elicitation by Kriegler et al. (2009). With exception of the unclear links, all other link strengths range from value 1 (or -1 for the negative link) to their maximum link strengths. From these ranges ensemble values are drawn as part of the Monte Carlo uncertainty propagation process. Setting the lower range of* s_{ij} *to (-) 1 avoids overweighting the case of no interaction (which would correspond to* $s_{ij} = 0$*) for links clearly expected to be positive or negative, in relation to the unclear links, where the range from -1 to 1, including 0, is explicitly part of the range. The possibility of no interaction is accounted for via the global interaction strength parameter* d *that is varied in 0.1 steps from 0 to 1 (see Eq. 1).*

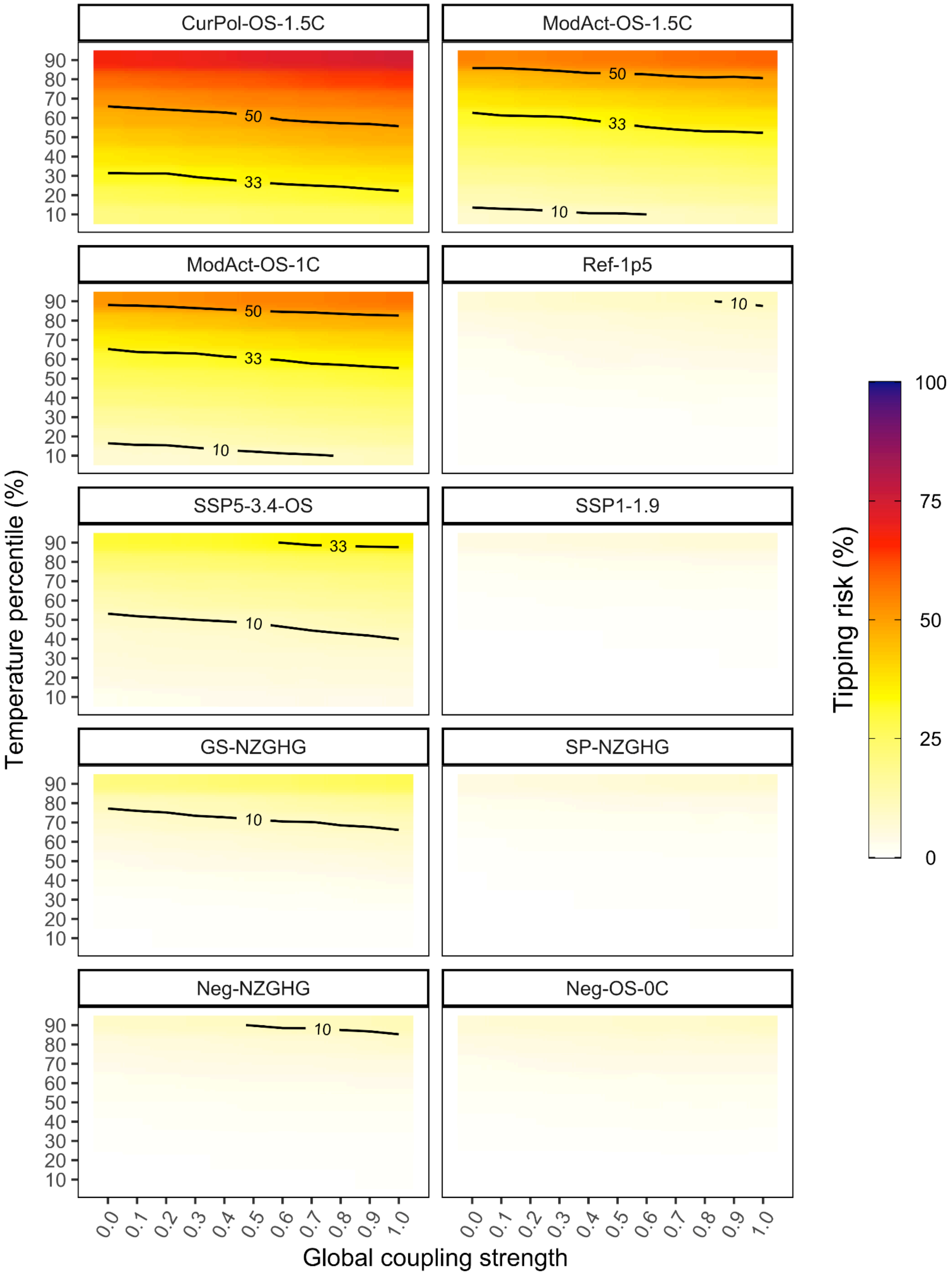

***Supplementary Fig. 3 | Medium-term risk dependent on global coupling strength**. The y-axis accounts for the uncertainties in the climate response. The x-axis accounts for the global coupling strength (see parameter d in Eq. (1)) that determines the ratio of individual subsystem dynamics (sole contribution when coupling strength 0) and interaction dynamics (equal contributions when coupling strength 1). Isolines for 10, 33, 50, 66, and 90 % tipping risk.*

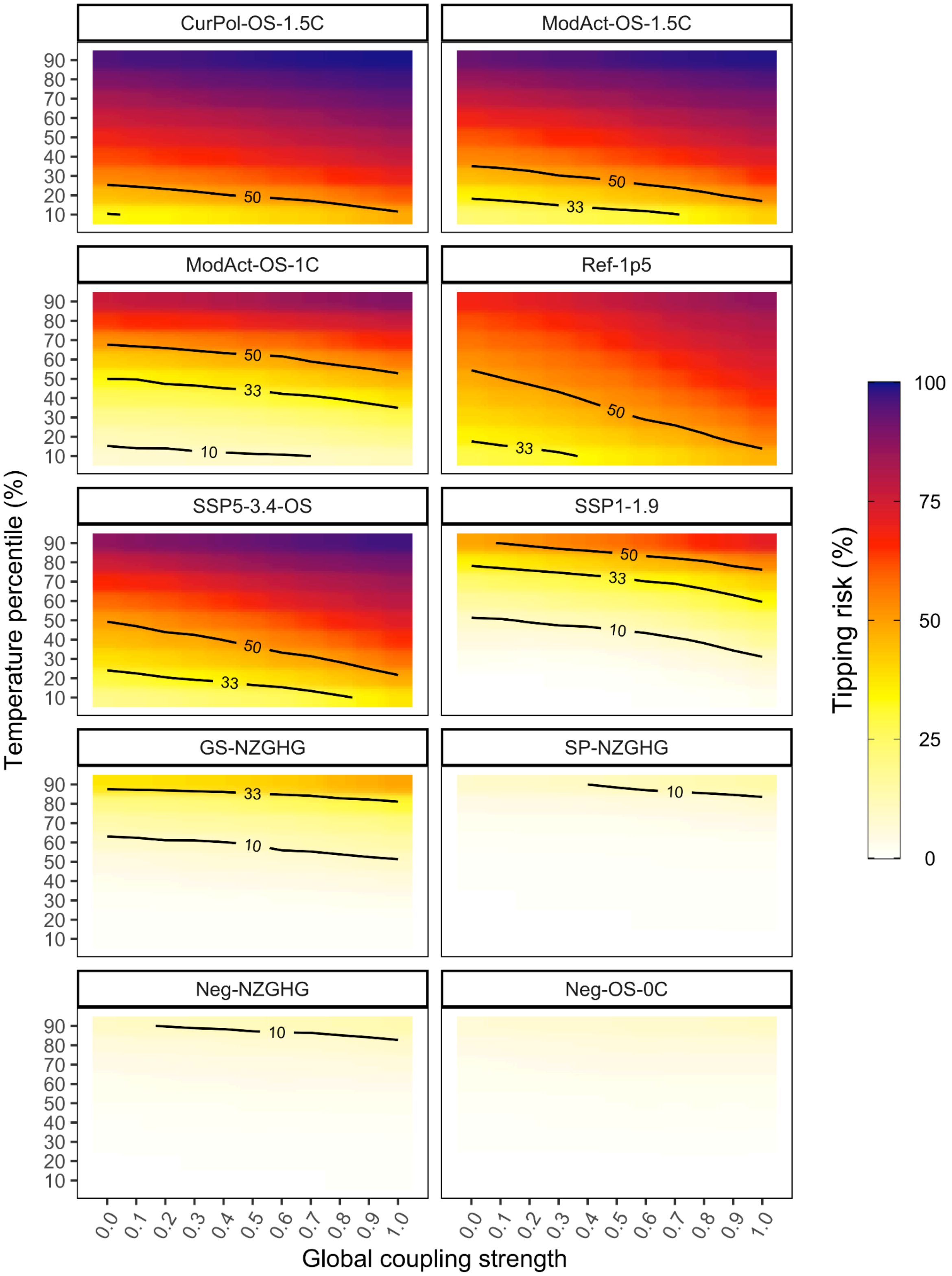

Supplementary Fig. 4 | Long-term risk dependent on global coupling strength. *The y-axis accounts for the uncertainties in the climate response. The x-axis accounts for the global coupling strength (see parameter d in Eq. (1)) that determines the ratio of individual subsystem dynamics (sole contribution when coupling strength 0) and interaction dynamics (equal contributions when coupling strength 1). Isolines for 10, 33, 50, and 66 % tipping risk.*

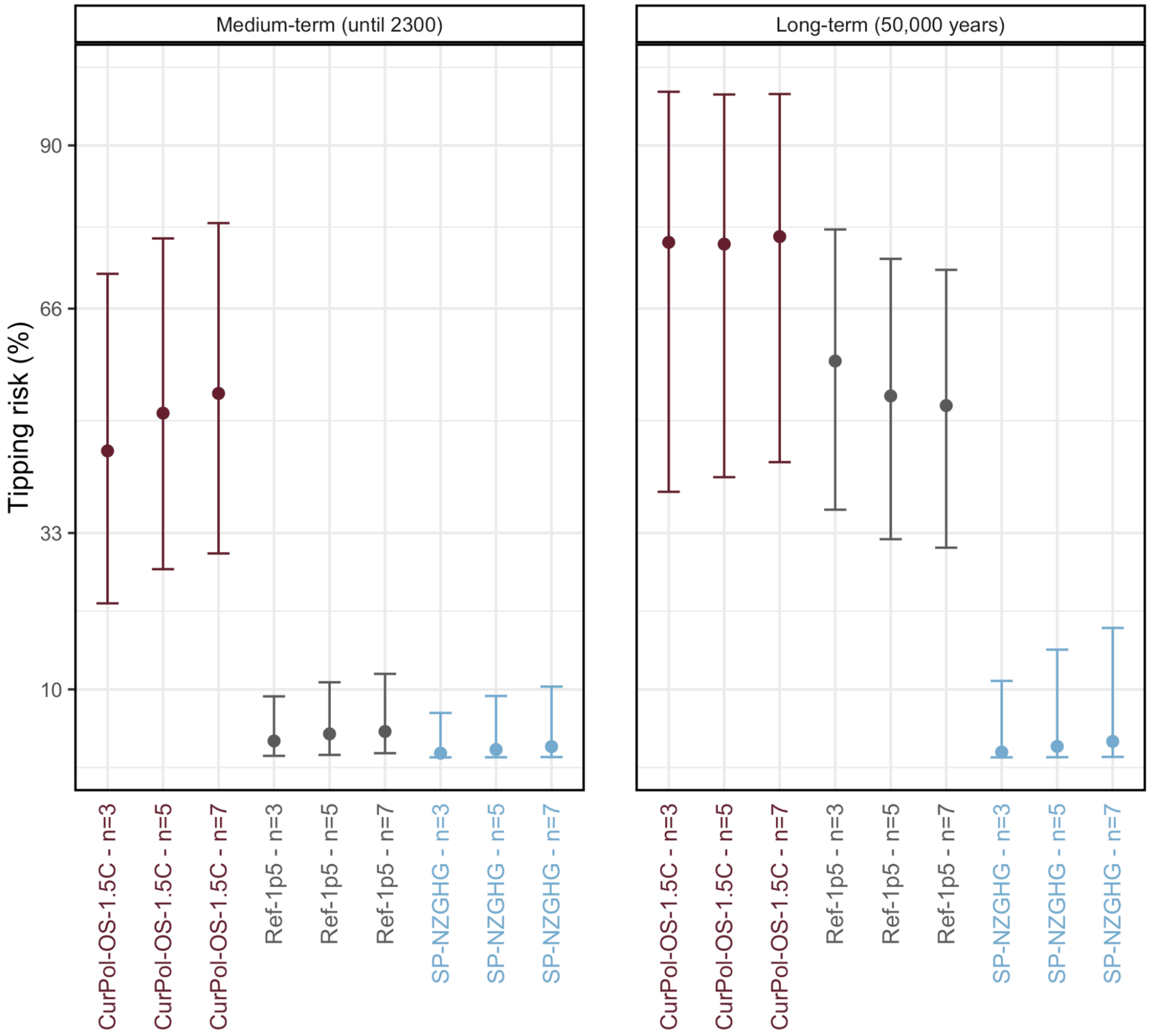

Supplementary Fig. 5 | Sensitivity analysis with respect to the exponent n. *Sensitivity analysis for different exponents n in Eq. 1.* ***a,*** *In the medium-term (until 2300) and* ***b,*** *in the long-term (50,000 years), with the probability derived from the median temperature trajectory as centre dots, and the range spanning the 10-90th temperature percentiles for three selected scenarios. In the main manuscript, the established model uses $n = 3$. Here, we additionally depict tipping risks for $n = 5$ and $n = 7$ (see Methods). Model runs with different values for n are shown to be in close agreement with each other.*

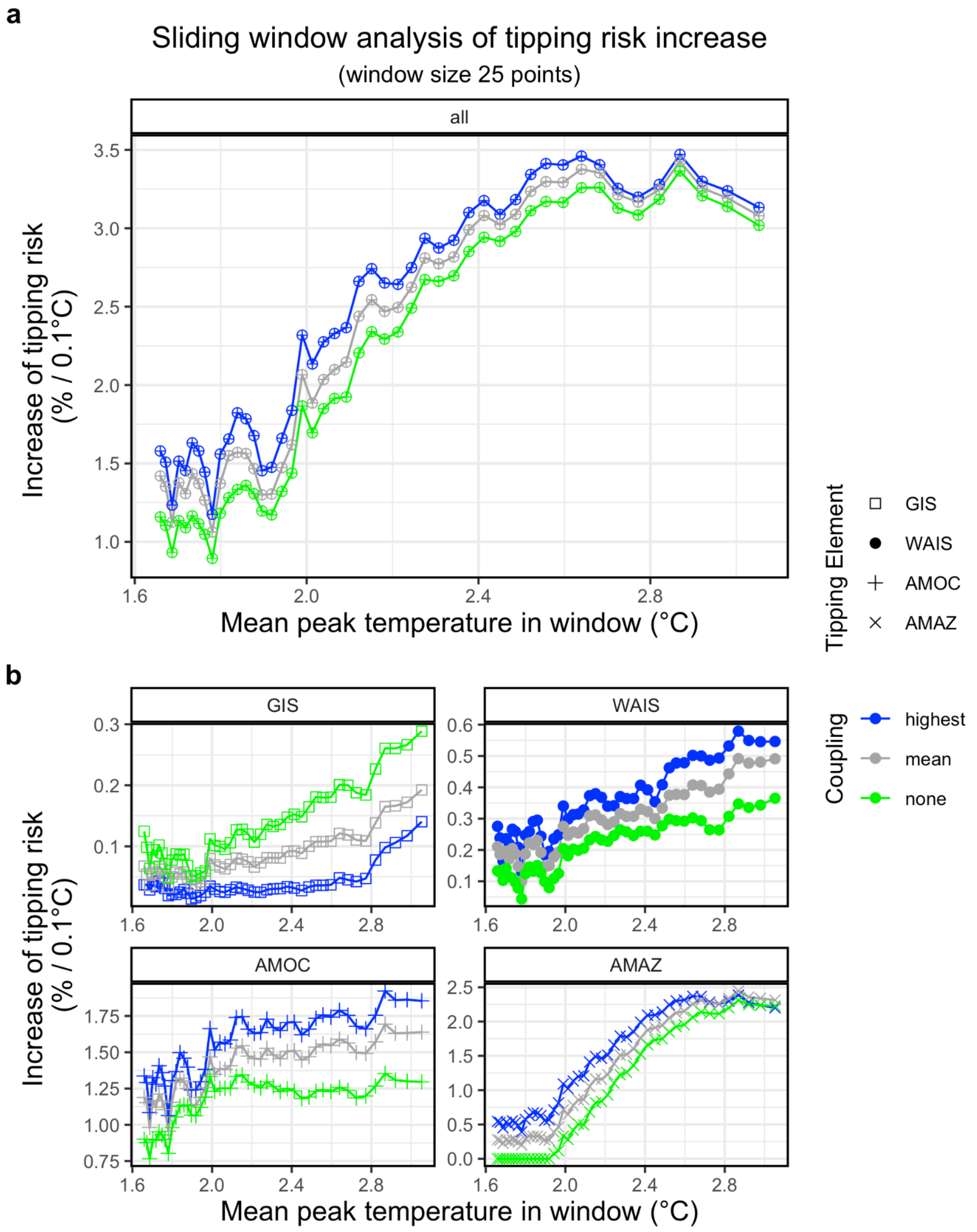

Supplementary Fig. 6 | Impact of coupling strength on non-linearity in tipping risk increase. *Each point denotes the slope of a linear fit through a window of 25 adjacent data points of overshoot peak temperature vs. tipping risk (see Figure 4a), representing the increase in tipping risk against the average overshoot peak temperature within this window. The tipping risk increase per window is shown* ***a,*** *across all tipping elements, as well as* ***b,*** *for all four tipping elements separately. Note that the y-axis is differently scaled in all subplots for better visibility. Green denotes no coupling, blue denotes highest coupling (global coupling strength set to 0.9), grey denotes the average across all global coupling strengths.*

Alternative metric: *Time-Averaged temperature increase during the OverShoot* (TA-OS)

An overshoot is typically characterised by duration as well as temperature exceedance above a predefined level. Since both of these variables are decisive for tipping risks[2], we have conducted additional analysis in which we quantify the overshoot above 1.5 °C via the *Time-Averaged temperature increase during the OverShoot* (TA-OS), defined by the warming during the overshoot averaged over the overshoot duration (see Supplementary Fig. 7) and tested the analysis performed against overshoot peak temperature in Fig. 4 and Supplementary Fig. 6 with this alternative metric. We find a similar non-linear acceleration in risk at 1.8 °C mean TA-OS (see Supplementary Fig.s 9b, 10) which corresponds to around 2.0 °C mean peak warming (see inset Supplementary Fig. 10b).

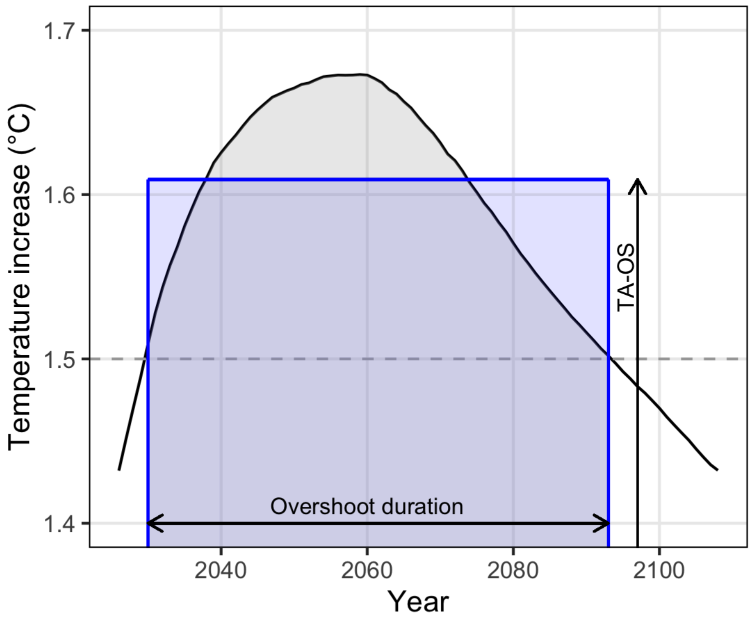

Supplementary Fig. 7 | Schematic figure. Time-averaged temperature increase during the overshoot above 1.5°C (TA-OS), *illustrated on the median temperature outcome of the Neg-NZGHG scenario. Temperature increase is given relative to preindustrial. The grey area under the curve corresponds to the area shaded blue and is obtained by dividing the integral of the temperature increase by the overshoot duration.*

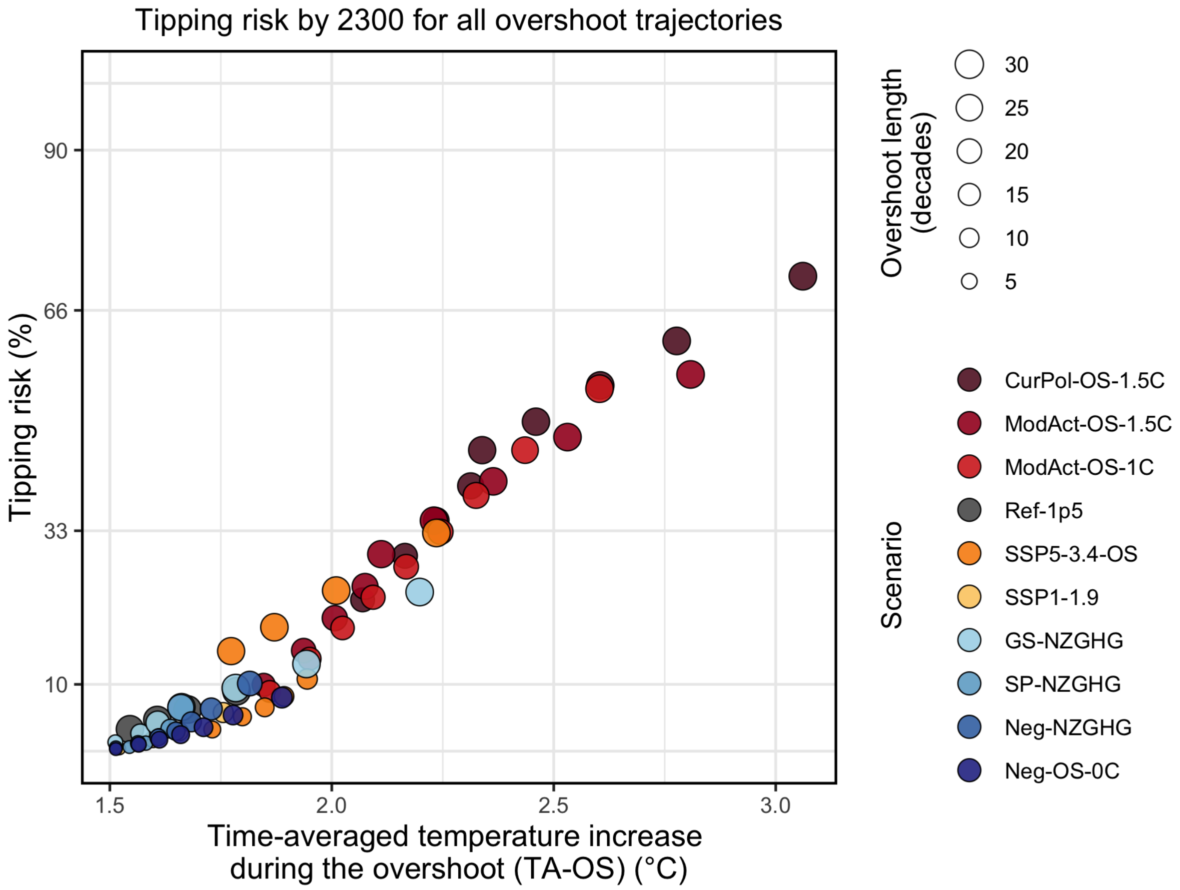

Supplementary Fig. 8 | Impact of magnitude and length of overshoot above 1.5 °C. *Increase in tipping risk (%) until 2300 per Time-averaged temperature increase during the overshoot above 1.5 °C (TA-OS), for all trajectories with overshoot above 1.5 °C. Each point represents one temperature percentile (10-90 %) of a scenario and is sized by the duration of the overshoot. Scenario colours allow for identification of the corresponding scenario.*

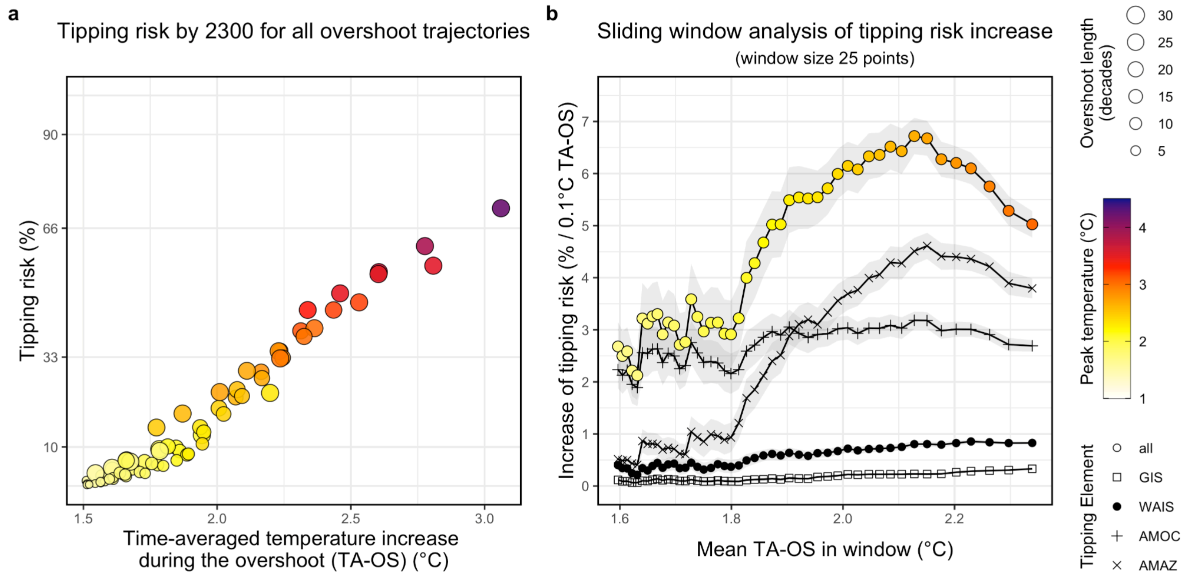

***Supplementary Fig. 9 | Impact of magnitude and length of overshoot above 1.5 °C and non-linear increase in tipping risk**. As Fig. 4, with Time-averaged temperature increase during the overshoot above 1.5 °C (TA-OS) on the x-axis (see Supplementary Figure 7). **a,** Increase in tipping risk (%) until 2300 per TA-OS, for all trajectories with overshoot above 1.5 °C. Each point represents one temperature percentile (10-90 %) of a scenario, is coloured by the peak temperature increase and sized by the duration of the overshoot. For the corresponding scenario information, see Supplementary Fig. 8. **b,** Each point represents the slope of a linear fit through a window of 25 adjacent data points of TA-OS vs. tipping risk (see panel a), thereby denoting the increase in tipping risk for this window, against the average amount of TA-OS within this window. The tipping risk increase per window is shown for all four tipping elements separately, as well as across all tipping elements, coloured by the average peak temperature increase of the respective sliding window and sized according to the average overshoot length. Shaded areas represent the 95 % confidence interval.*

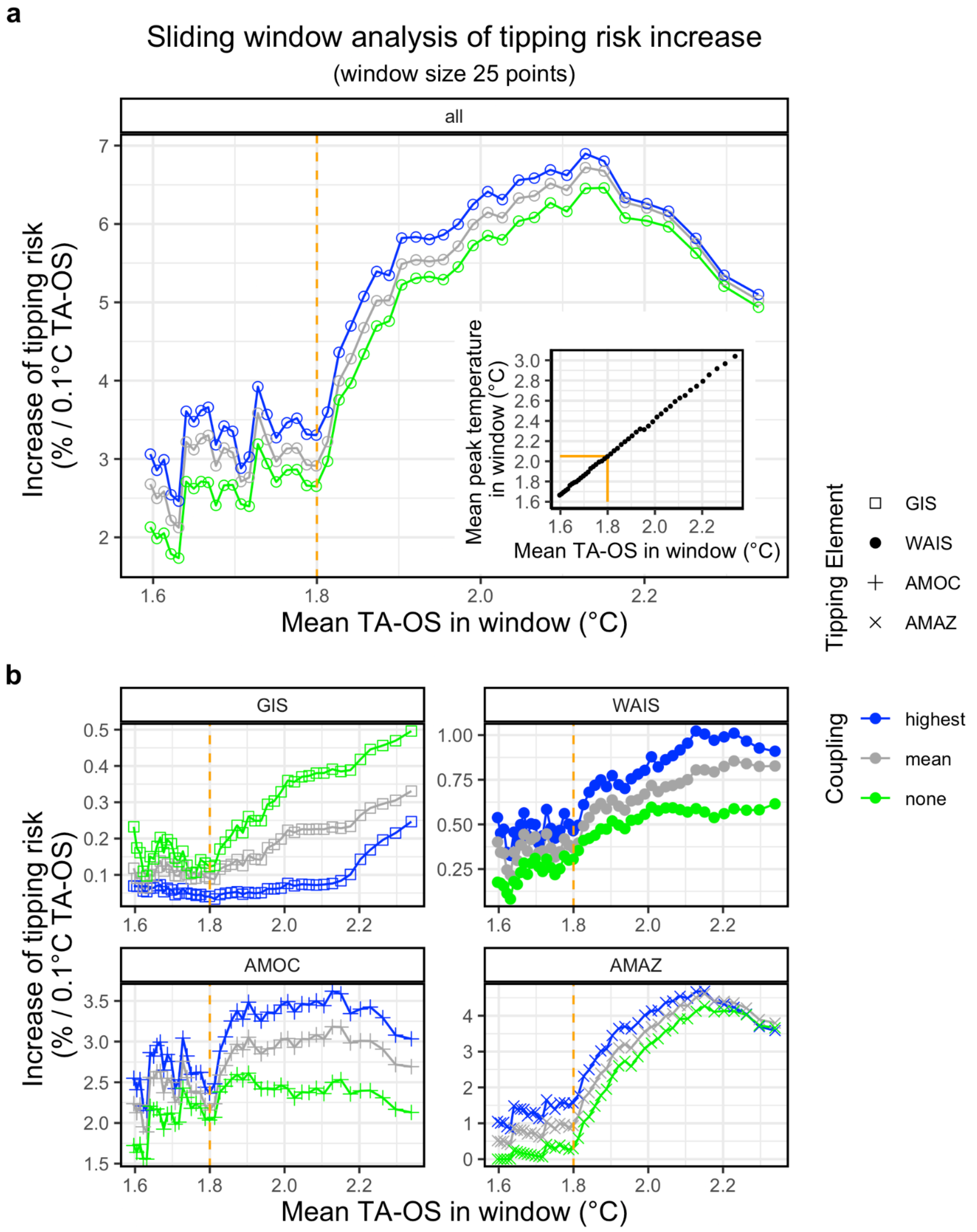

Supplementary Fig. 10 | Impact of coupling strength on non-linearity in tipping risk increase. *Each point denotes the slope of a linear fit through a window of 25 adjacent data points of Time-averaged temperature increase during the overshoot above 1.5 °C (TA-OS) vs. tipping risk (see Supplementary Fig.s 8, 9a), representing the increase in tipping risk against the mean TA-OS within this window. The tipping risk increase per window is shown* ***a,*** *across all tipping elements, as well as* ***b,*** *for all four tipping elements separately. The orange dashed line is at 1.8 °C TA-OS. Green denotes no coupling, blue denotes highest coupling (global coupling strength set to 0.9), grey denotes the average across all global coupling strengths. Note that the y-axis is differently scaled in all subplots for better visibility.* ***The inset in a*** *shows the relationship between the mean peak temperature and mean TA-OS per window, with orange lines highlighting the correspondence of 1.8 °C TA-OS with ~2 °C mean peak temperature.*

Supplementary References

1. Bochow, N. *et al.* Overshooting the critical threshold for the Greenland ice sheet. *Nature* **622**, 528–536 (2023).
2. Wunderling, N. *et al.* Global warming overshoots increase risks of climate tipping cascades in a network model. *Nat. Clim. Change* **13**, 75–82 (2023).
